# Different strength declines in leg primary movers versus stabilizers across age—Implications for the risk of falls in older adults?

**DOI:** 10.1371/journal.pone.0213361

**Published:** 2019-03-07

**Authors:** Franziska Daun, Armin Kibele

**Affiliations:** Institute for Sports and Sport Science, University of Kassel, Germany; University of L'Aquila, ITALY

## Abstract

This study investigated differences in the declines of isometric strength in hip abductors and adductors versus knee extensors across four different age groups (n = 31: 11.2 ± 1.0 y, n = 30: 23.1 ± 2.7 y, n = 27: 48.9 ± 4.4 y, and n = 33: 70.1 ± 4.2 y) with a total of 121 female subjects. As a starting point, we assumed that, during their daily activities, elderly people would use their leg stabilizers less frequently than their leg primary movers as compared to younger people. Given that muscle strength decreases in the course of the aging process, we hypothesized that larger strength declines in hip abductors and hip adductors as compared to knee extensors would be detected across age. Maximal isometric force for these muscle groups was assessed with a digital hand-held dynamometer. Measurements were taken at 75% of the thigh or shank length and expressed relative to body weight and lever arm length. Intratester reliability of the normalized maximal torques was estimated by using Cronbach’s alpha and calculated to be larger than 0.95. The obtained results indicate a clearly more pronounced strength decline in hip abductors and hip adductors across age than in the knee extensors. Therefore, a particular need for strength training of the lower extremity stabilizer muscles during the aging process is implied.

## Introduction

The decline in motor performance is a prominent characteristic of human aging. It is considered a major contributor to the risk of falls in the elderly. In particular, decreases in muscle strength [1–3], force steadiness [4–6], or power that a muscle can produce [7–10] have been identified in increasing the probability for falls when aging. Lower limb resistance training alone or in combination with balance training has been recommended to counteract this risk [11–15]. For example, Persch and co-workers [12] showed in a randomized, controlled study that lower limb strength training improved fall-related gait kinematics. In particular, gait speed parameters have been identified to benefit from physical exercise including strength training regimens [11,16–18] with preferred gait speed to be more sensitive to exercise programs than maximal gait speed [19]. Among the most prominent causes of strength loss, decreases in the muscle mass and the neuromuscular function have been reported [20–23].

Resistance training has been suggested to elicit effective countermeasures in elderly individuals to evoke muscle hypertrophy along with substantial changes in neuromuscular function, respectively [24]. Exercise-related increases in the muscle mass were reported even for old subjects beyond 75 years of age [25]. However, findings are inconclusive as to which type of resistance training is most effective for the elderly population. While some authors [21,26,27] advocate high intensity training above 60 percent of the 1RM to be more beneficial for strength improvements in the elderly other studies promote low intensity resistance training for that age group [18,28]. In addition, findings suggest that high intensity resistance training may be effective for improving strength but does not improve force steadiness in sub-maximal isometric contractions [29].

In the literature, age-related decreases in strength have been well-documented in numerous studies. To quantify the yearly decline in strength in the elderly, many studies have used differences between young and old subject groups in cross-sectional studies or observed strength losses in aging individuals in longitudinal studies over time. There are indications that cross-sectional data may underestimate true aging-related strength losses [22,30]. Recently, Maden-Wilkinson and co-workers [31] found maximal isometric strength values in the knee extensors of roughly 70-year olds only reaching 60 percent of the values found in roughly 20-year old participants evolving to a strength loss of 12 percent per decade. Overall, and starting slowly at approximately 30 years of age, declines in strength are estimated to be 1 to 2 percent per year independent of the gender [20,32,33]. Beyond 60 years of age, the loss of strength appears to increase more rapidly, but eventually levels off in the seventh and eighth decades.

Studies assessing changes in muscle mass and strength in the same sample reported a loss of strength 2 to 5 times faster than loss of muscle mass [22]. Here, muscles in both the upper and lower limbs, including proximal and distal locations, were examined. Furthermore, while some authors consider the observed strength decreases to be similar across all muscles [20,32], other researchers found lower limb strength to be lost more rapidly than upper limb strength [30,34].

Among the lower limb muscles analyzed, when comparing old with young subjects’, knee extensors and hip muscles have been evaluated most frequently. For example, Frontera and Bigard [33] reported that knee extensor and flexor strength declines in longitudinal studies of approximately 1 to 2 percent per year. In a study by Johnson and co-workers [35], age-related changes in hip abductor muscles and adductor muscles were analyzed using maximal isometric torques and isokinetic torques in young and older female subjects with mean ages of 23 and 74 years. For the isometric measurements, average decreases of 34 percent in the abductors and 24 percent for the adductors were reported which averages to a yearly decrease of roughly 0.7 percent and 0.5 percent. For the isokinetic measurements, larger yearly decreases of 0.9 percent and 1.1 percent were found. For the hip extensor and flexors, Dean and colleagues [36] identified 31 percent and 22 percent lower maximal isometric torques in similar age groups of, on the average, 23-year vs. 74-year old women. Thus, a yearly decrease of 0.6 percent and 0.4 percent was found. Similarly, Morcelli et al. [37] found 49 percent lower peak torques in the hip extensors and 42 percent for the hip flexors of 67-year old women as compared to 21-year old women (1.1 percent and 0.9 percent decrease per year). In a previous study, Morcelli et al. [38] had found 9, 12 and 13 percent lower strength during hip extension, abduction, and adduction in old fallers as compared to old non-fallers with an average age of 69 and 66 years.

Summarizing the above results, there is conclusive evidence that the strength capacity of human subjects is decreasing throughout the aging process. However, results are inconclusive about the amount of decrease and whether this amount is found for all skeletal muscles in the same way. In this respect, using a retrospective analysis, Trudelle-Jackson and coworkers [39] found inconsistent strength declines across four age groups for different muscles when comparing lower extremity strength measurements of 240 women aged 50–89 years. Strength declined significantly with age in all muscle groups except the knee extensors. Older Women had less hip flexor or abductor strength and were more likely to fall. Therefore, a closer look at the age-related decline in muscles fulfilling different functional purposes for postural control and locomotion was in need. More specifically, regarding the risk of falls in elderly people, it is of primary interest to find out whether stabilizing muscles and locomotive muscles would show different amounts of strength loss during the aging process. While primary movers accomplish mostly locomotive tasks, stabilizer muscles are considered to contribute to joint stiffening through co-contractions while showing an early activation onset in response to perturbation using feed-forward and/or feedback control processes [40].

From a naïve standpoint, elderly people may be expected to still regularly use their lower extremity primary movers during their daily tasks such as walking upstairs to the bedroom or going shopping. Therefore, at least some degree of daily primary muscle activation may be expected. However, it appears questionable whether the lower exetremity stabilizer muscles would be activated to a similar degree as seniors are usually not exposing themselves frequently to unstable situations during daily activity. In this regard, Kibele [41] has pointed out higher levels of stabilizer muscle activation when exercising on unstable versus stable platforms. If lower extremity stabilizer muscles are not regularly used, they would be expected to show larger age-related declines than primary movers. Therefore, it was the goal of this study to compare the strength values of lower extremity stabilizers to primary movers across different age groups. We hypothesized that a systematic decline in this ratio should be observed when comparing young, middle-aged, and older subjects identifying a more pronounced strength loss in the lower extremity stabilizer muscles. As a reference, 10-year old children were examined as well to find out about a possible curvature across age groups in the lower extremity stabilizer to primary mover strength ratio.

Maximal isometric torques were measured for the knee extensors and the hip adductor plus the hip abductors in a cross-sectional study with age categories: 10- to 13-year old children and 19- to 29-, 41- to 55-, and 63- to 79-year old adults. To avoid any gender-related bias, only female subjects were included in the study. In the literature, strength declines across age are reported to evolve in the same way for male and female adults when controlling muscle mass [20,32].

## Materials and methods

The study was approved by the local ethics committee of the University of Kassel as part of a fall prevention trial (E052016058). The study complies with the ethical standards of the latest Declaration of Helsinki (WMA, Oct. 20132)

### Subjects

A total of 121 randomly selected female subjects were recruited for the study. The subjects had to meet the criteria of being healthy with a body-mass-index less than 30 and not suffering from diseases within the neuromuscular system. For the justification of the sample size, a two-step approach was adopted.

It has become a common requirement in the author guidelines of many scientific journals to justify sample size through a power analysis according to the seminal work of Jacob Cohen [42]. This value can be calculated through the functional interdependence with the α-error, the power, and the effect size of a given statistical test. While default recommendations for the α-error and the power have evolved in classical statistics over the years and settled on α = 0.05 and power = (1–4α) [42], priori effect size estimates are less clear to derive for a sample size calculation. The options are to either use a medium effect size value according to Cohen [42] or to derive the effect size from a previous study using the same statistics [43]. While studies on strength differences between lower extremity muscles across age groups are so far amiss in the literature, and a medium effect size according to Cohen [42] as a best guess did not appear to be a well-founded solution, a two-step approach with a pilot study was adopted to achieve a reasonable effect size for the sample size calculations within a power analysis.

The study was subdivided in a first part with strength measurements across the four age groups with rule of thumb sample sizes between 15 and 20 subjects, as subjects were examined in a non-systematic order independent of their age. These measurements were conducted by a female experimenter. Using the effect sizes of this first assessment of the ratios between normalized abductor to knee extensor strength and the normalized adductor to knee extensor strength, a power-analysis was conducted using the G*Power software package [44] with a α-error of 0.05 and a power of 0.80 [42]. As a result, above medium effect size values of 0.32 and 0.39 were obtained. Hence, with an estimated effect size of 0.3, a G*power sample size calculation of 120 subjects total was found. Therefore, the original groups sample sizes were increased to approximately 30 subjects per goup. For the extended assessment, a second female experimenter was deployed to increase measurement objectivity.

In total, all subjects were grouped according to their age. The demographic and anthropometric identifiers of the different groups are listed in Table 1. The first age group (AG10) was composed of 31 young girls aged 10–13 years who were recruited from two local grade schools. Written consent was provided by the school and by the legal guardians of the children. The second age group (AG25) consisted of 30 physically active young women aged 19–29 years who were recruited among the female sport science students at the Institute for Sports and Sport Science at the University of Kassel. The third age group (AG50) encompassed 27 physically active middle-aged between 41 and 55 years of age recruited through fitness courses in nearby studios or through adult education courses. The fourth age group (AG70) comprised a total of 33 healthy elderly women aged 65–79 years recruited from senior citizen organizations within the city of Kassel and its vicinity. None of the subjects from AG70 was engaging in regular physical exercise.

**Table 1:** Mean values and standard deviations of demographic and anthropometric data if the four age groups (AG10 = 10- to 13-year old girls, AG25 = 19- to 29-year old females, AG50 = 41- to 55-year old females, and AG70 = 63- to 79-year old females) including the duration of physical activity per week. Values in parenthesis indicate the results of the first assessment.

	Age Groups (Mean ± SD)			
	AG10 *n* = 31	AG25 *n* = 30	AG50 *n* = 27	AG70 *n* = 33
	*(AG10* *n = 16)*	*(AG25* *n = 15)*	*(AG50* *n = 15)*	*(AG70*: *n = 20)*
Age	11.2 ± 1.0 *(10*.*6 ± 0*,*5)*	23.1 ± 2.7 *(23*.*9 ± 2*.*6)*	48.9 ± 4.4 *(48*.*4 ± 5*.*1)*	70.1 ± 4.2 *(71*.*8 ± 4*.*5*
Body Height [cm]	154.2 ± 9.2 *(149*.*0 ± 7*.*0)*	166.3 ± 6.9 *(165*.*9 ± 7*.*4)*	168.8 ± 5.2 *(170*.*3 ± 4*.*5)*	162.7 ± 6.9 *(161*.*1 ± 5*.*1)*
Body Weight [kg]	45.1 ± 9.4 *(40*.*0 ± 7*.*3)*	62.4 ± 8.3 *(59*.*7 ± 6*.*8)*	67.9 ± 8.4 *(67*.*9 ± 8*.*5)*	65.9 ± 8.3 *(65*.*9 ± 6*.*4)*
Body-Mass-Index [kg/m^2^]	18.8 ± 2.6 *(17*.*9 ± 2*.*3)*	22.5 ± 2.1 *(21*.*6 ± 1*.*4)*	23.8 ± 2.6 *(23*.*4 ± 2*.*8)*	24.9 ± 2.7 *(25*.*4 ± 1*.*9)*
Femur Length [m]	37.8 ± 3.3 *(36*.*6 ± 2*.*1)*	42.4 ± 4.0 *(40*.*8 ± 2*.*8)*	41.2 ± 3.2 *(42*.*4 ± 2*.*3)*	40.4 ± 2.4 *(40*.*3 ± 2*.*4)*
Shank Length [m]	37.8 ± 2.2 *(36*.*9 ± 2*.*3)*	39.2 ± 2.6 *(40*.*3 ± 2*.*6)*	40.9 ± 2.2 *(41*.*7 ± 1*.*7)*	39.4 ± 2.5 *(39*.*5 ± 1*.*9)*
Physical Activity [min/week]	354 ± 140 *(316 ± 48)*	430 ± 262 *(410 ± 171)*	186 ± 161 *(156 ± 79)*	71 ± 100 *(64 ± 67)*

For all participants, the duration of physical activity per week was assessed through the German short version of the Freiburg Physical Activity Questionnaire [45] in conjunction with some standardized open questions. Prior to testing, all subjects were thoroughly informed about the measurement procedures. They were also requested to sign an informed consent on their participation and on the anonymous usage of their data for researching purposes. For the young girls, these forms were signed by the legal guardians.

### Materials

To examine the maximal isometric strength in the knee extensors, the hip abductors, and the hip adductors, the Lafayette Manual Muscle Testing System (LMMTS) (Type 01165, Lafayette Instrument Company, Lafayette, Indiana, United States) was used. This hand-held dynamometer measures a range of 0 to 1335 N with an accuracy of ± 1 percent.

### Procedures

Most testing was conducted in the biomechanics laboratory at the Sports Institute of the University of Kassel. The testing of the 10-year old girls was carried out in two schools in the city of Kassel. The data collection for each subject took 35 to 45 minutes.

After signing the written informed consent on the participation in the study, subjects were asked about their age, occupational activities, and general daily physical activities. Furthermore, they were interviewed on their engagement in regular sports and the duration of exercise. The body height and the body weight were then assessed through a standard tape measure and a standard body scale with an accuracy of 0.1 kg. In order to identify the dominant leg, the subjects were asked to imagine kicking a ball as far as possible. We provided the group of young girls with a ball, so they were able to try out which was their preferred leg.

In order to locate the exact position for the LMMTS for the torque calculations, the lengths of the femur and the shank length were previously assessed while the subjects were seated on a bench with their lower legs hanging loose above the ground. The femur length was taken as the distance between the highest point of the trochanter major and the lateral knee joint gap between the femur and the fibula head. The shank length was measured from the lateral knee gap to the highest point of the lateral malleolus. For the comparison of the experimental groups, we calculated 75 percent of the total length for each leg segment and marked the points for the application of the top edge of the hand-held dynamometer [46].

Prior to the strength measurements, the subjects were instructed to immediately report any feeling of discomfort during the measurements possibly influencing strength development. To become accustomed to the measurement device, subjects performed a submaximal practice trial for each muscle group. Three measurements per muscle group were conducted for the left and the right leg. The instructions as well as the verbal encouragements were standardized to ensure experimenter objectivity [47,48]. To achieve maximal torque values, subjects were instructed to apply a steady maximal force against the pad of the hand-held dynamometer for at least three seconds until the experimenter’s stop signal. The make-test method was adopted with subjects applying a steady force to the hand-held dynamometer while the experimenter was trying not to move it. The reliability of the make-test has been shown to be higher than in the break-test method where the experimenter attempts to overcome the force that the subject applies to the dynamometer [49]. The three measurements for each muscle group were separated by resting periods lasting for at least one minute to eliminate local fatigue [50]. The results of the measurements were not fed back to the subjects during the testing procedure.

For the LMMTS measurements, the testing instructions from Lafayette Instrument Company were followed. In the literature, good to excellent reliabilities were reported when using the LMMTS to measure isometric strength exerted against a resistance produced by the experimenter [51]. In any case of deviations from the standardized testing positions, the measurement was repeated and correctional instructions were given to the subjects. The testing of the hip abductors and hip adductors were conducted first with a random selection of the muscle group tested. For these measurements, subjects were lying in a lateral position on a padded massage table. They were instructed to hold on to the outer edge of the massage table with the hand of the downward body side to minimize any rotation of the trunk about the longitudinal body axis during the measurements. During the testing of the hip abductors and hip adductors, the hip-trunk angle of the active leg was kept at 0 degrees providing a neutral position of the hip in the sagittal plane while the knee joint angle of the active leg was 180 degrees [52]. To avoid any interference during testing, the inactive leg was placed with an 80 to 90 degrees knee joint angle next to the active leg. The subjects were instructed to pull the toes of the active leg upwards for a dorsal extension of the foot to avoid co-contractions of other muscles groups and isolate the muscle groups tested for a maximal strength development [47,53].

For measuring the maximal isometric strength of the hip abductors, the subjects maintained a side-lying position with a neutral hip position in the sagittal plane but with the bottom leg bent and the top leg elongated. Again, the subjects were instructed to pull the toes of the test leg upwards. In contrast to the testing of the hip adductors, the subjects were instructed to lift the test leg from the negative angular position in the frontal plane immediately before the actual strength testing to enable measuring the maximal isometric strength of the hip abductors at a 0-degree angle.

To measure the maximal isometric strength of the knee extensors, the subjects sat upright and with folded arms on the padded massage table covered with a nonskid surface which prevented any rotation along the longitudinal body axis. The knee joint angle as well as the hip joint angle amounted to 90 degrees each. The subjects were instructed to remain in the upright seated position throughout the measurement to avoid any contribution of the hip extensors or any kind of pushing by using the body weight. They were able to move their knee joint freely without experiencing any contact with the ground. To provide a maximal resistance during testing, the experimenter positioned herself backwards supported against a wall directly in front of the subjects.

### Data collection and statistical analysis

For the statistical data evaluation individual means across three measurements were collected as group mean values and standard derivation for each parameter. The normal distribution of the data was assessed by the Shapiro-Wilks test. Variance homogeneity was analyzed by the Levene-test. Furthermore, the reliability of the measurements for the maximal isometric forces was evaluated by Cronbach’s alpha.

To calculate the normalized maximal isometric torques (NMT in Nm), the measured maximal isometric force recordings (in N) were multiplied by the measured lever arms (in m). The values were then normalized by body weight [42,54,55,56]. To analyze the hypothesized difference in the strength decline between leg primary movers and stabilizers across age, ratios were calculated between NMTs in the hip abductors and the hip adductors vs the knee extensors. In addition, the NMT ratios of hip abductors to hip adductors were assessed.

A one-way analysis of variance with SPSS V23.0 was used to analyze differences in the mean values of the normalized strength values and their ratios between age groups. For a violation of the variance homogeneity, the Welch test was applied. Effect sizes for group mean differences were estimated by the ω^2^-values (small effects for 0 < ω^2^ < = 0.06; medium effects for 0.06 < ω^2^ < = 0.14, and large effects for 0.14 < ω^2^) which are assumed to provide the least bias [57,58]. In addition, Cohen’s f effect size [42] (small effects for f = 0.1, medium effects for f = 0.25, and large effects for f = 0.4) was calculated for the sample size estimation in the G*Power analysis [44]. The Tukey-HSD post-hoc test was conducted to analyze pairwise differences between the group means with given variance homogeneity. For a violation of the variance homogeneity, the Games-Howell post-hoc test was used. The Kruskal-Wallis-Test with the Dunn-Bonferroni post-hoc test was applied for non-parametric testing to confirm the results of the parametric testing procedures. Overall, the level significance was p < 0.05 and for highly significant differences p < 0.01.

## Results

**Fig 1:**
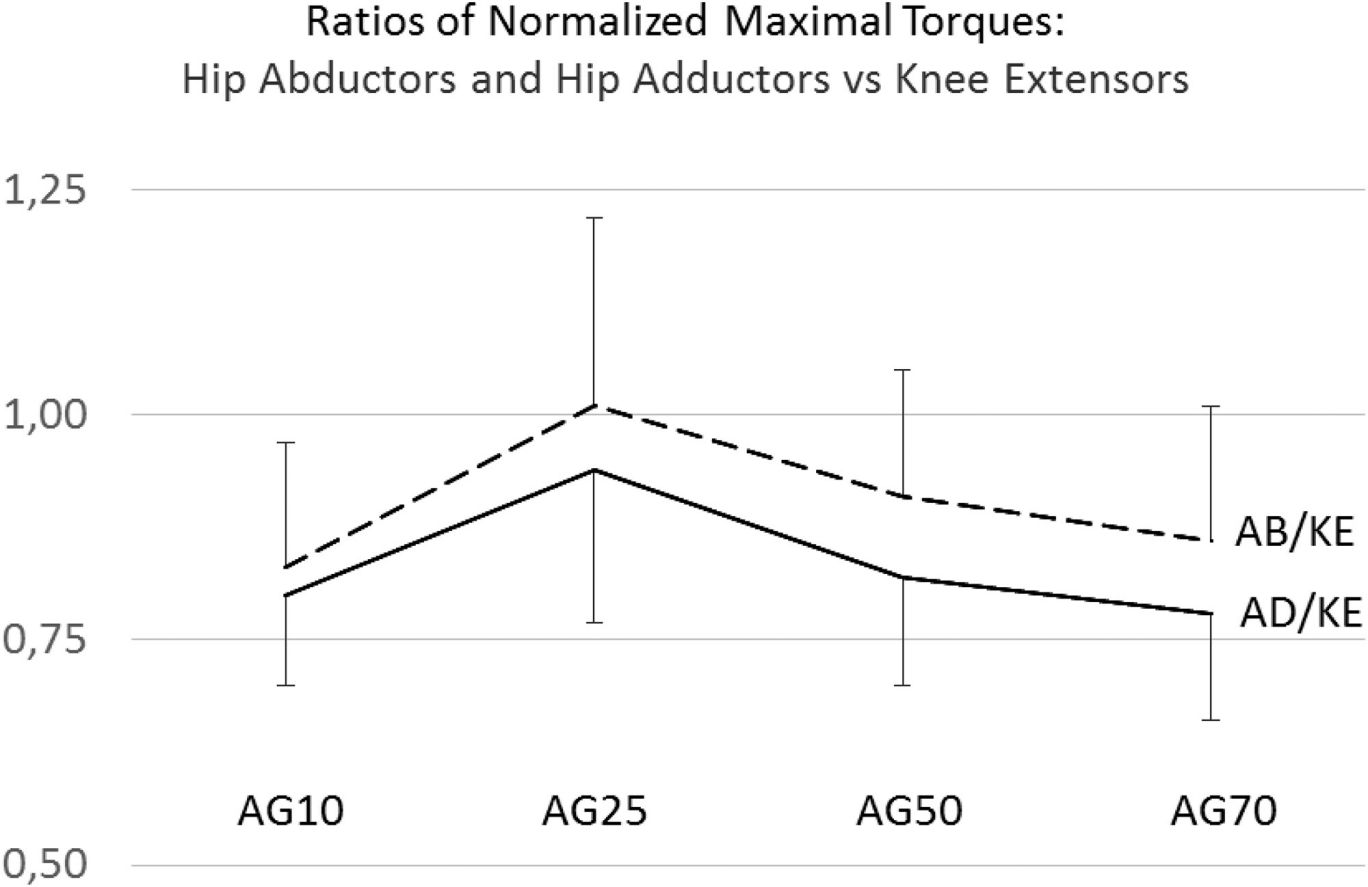
Normalized maximal torques in the knee extensors (KE = solid line), the hip abductors (AB = dashed line), and the hip adductors (AD = dashdotted line) in the four age groups (AG10 = 10- to 13-year old girls, AG25 = 19- to 29-year old females, AG50 = 41- to 55-year old females, and AG70 = 63- to 79-year old females).

**Fig 2:**
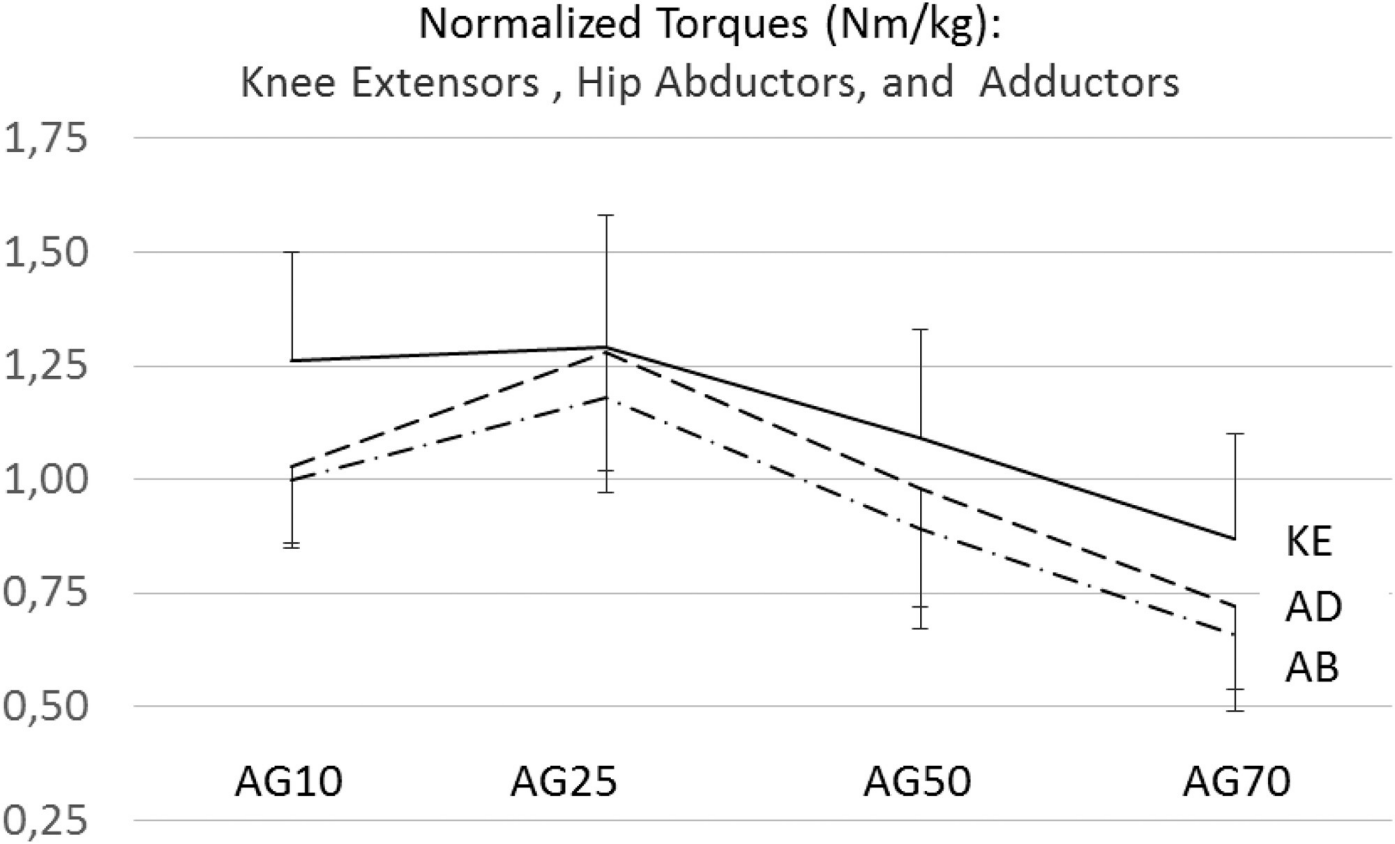
Ratios of normalized maximal torque ratios between the hip abductors and the knee extensors (AB-KE) (dashed line) and the hip adductors and the knee extensors (AD-KE) (dashed line) in the four age groups (AG10 = 10- to 13-year old girls, AG25 = 19- to 29-year old females, AG50 = 41- to 55-year old females, and AG70 = 63- to 79-year old females).

All normalized maximal torque (NMT) values used for the statistical data evaluation across age groups were found to be normally distributed. Self-reported physical activity times were not normally distributed within the age groups. Hence, a Kruskal-Wallis test was conducted to show highly significant differences in the physical activity times between the groups (Table 1). While AG25 showed largest physical activity times, the least physical activity per week was detected in AG70. The Cronbach’s alpha reliabilities across the three measurement repetitions for the maximal isometric force raw scores were found to be 0.97 for the knee extensors, 0.98 for the hip abductors, and 0.97 for the hip adductors. The group mean values and standard deviatons for the normalized maximal isomtric torques (NMT) and the group mean values of the NMT ratios between the hip abductors and the hip adductors to the knee extensors are provided in Table 2. A graphical illustration of these results is shown in Figs 1 and 2. A significant violation of the variance homogeneity assumption was detected for the NMTs in the hip abductors therefore the Welch test was applied. Highly significant differences between the groups with large effect sizes were detected for the NMTs produced by the hip abductors (F (3,62.2) = 34.88, p < 0.01; ω^2^ = 0.44), the hip adductors (F (3,117) = 39.39, p < 0.011; ω^2^ = 0.49), and the knee extensors (F (3,117) = 19.33, p < 0.01; ω^2^ = 0.31).

**Table 2:** Group mean values and standard deviation of the normalized maximal torques (NMT) in the knee extensors (KE), the hip abductors (AB), and the hip adductors (AD) as well as for the ratios between hip abductors vs hip adductors vs knee extensors in the four age groups (AG10 = 10- to 13-year old girls, AG25 = 19- to 29-year old females, AG50 = 41- to 55-year old females, and AG70 = 63- to 79-year old females). Values in parenthesis indicate the results of the first assessment.

Groups Variable	AG10 n = 31 *(AG10* *n = 16)*	AG25 n = 30 *(AG25* *n = 15)*	AG50 n = 27 *(AG50* *n = 15)*	AG70 n = 33 *(AG70* *n = 20)*	F-value	Effect Size f	Effect Size ω^2^
NMT AB [Nm/kg]	1.03 ± 0.17 *(1*.*09 ± 0*.*17)*	1.28 ± 0.26 *(1*.*39 ± 0*.*20)*	0.98 ± 0.26 *(1*.*12 ± 0*.*18)*	0.72 ± 0.18 *(0*.*74 ± 0*.*14)*	33.17**^1^ *(40*.35**)	0,97 *(1*,*47)*	0,44 *(0*,*64)*
NMT AD [Nm/kg]	1.00 ± 0.15 *(1*.*01 ± 0*.*11)*	1.18 ± 0.21 *(1*.*11 ± 0*.*11)*	0.89 ± 0.22 *(0*.*85 ± 0*.*16)*	0.66 ± 0.17 *(0*.*59 ± 0*.*15)*	39.39** (49.18**)	0,89 *(1*,*30)*	0,49 *(0*,*69)*
NMT KE [Nm/kg]	1.26 ± 0.24 *(1*.*35 ± 0*.*19)*	1.29 ± 0.29 *(1*.*51 ± 0*.*18)*	1.09 ± 0.24 *(1*.*22 ± 0*.*16)*	0.87 ± 0.23 *(0*.*89 ± 0*.*24)*	19.33** (32.29**)	0,68 *(1*,*20)*	0,31 *(0*,*59)*
NMT-Ratio: AB/KE	0.83 ± 0.14 *(0*.*81 ± 0*.*09)*	± 0.21 *(0*.*92 ±0*.*10)*	0.91 ± 0.14 *(0*.*93 ± 0*.*13)*	0.86 ± 0.15 *(0*.*87 ± 0*.*16)*	8.24**^1^ *(2*.89*)	0,45 *(0*,*32)*	0,15 *(0*,*08)*
NMT-Ratio: AD/KE	0.80 ± 0.10 *(0*.*75 ± 0*.*07)*	0.94 ± 0.17 *(0*.*74 ± 0*.*08)*	0.82 ± 0.12 *(0*.*71 ± 0*.*13)*	0.78 ± 0.12 *(0*.*67 ± 0*.*10)*	8.01**^1^ *(2*.*37 n*.*s*.*)*	0,46 *(0*,*39)*	0,15 *(0*,*06)*
NMT-Ratio: AB/AD	1.04 ± 0.07 *(1*.*08 ± 0*.*10)*	1.08 ± 0.07 *(1*.*25 ± 0*.*16)*	1.10 ± 0.09 *(1*.*34 ± 0*.*24)*	1.09 ± 0.10 *(1*.*29 ± 0*.*18)*	4.15**^1^ *(6*.64**)	0,32 *(0*,*58)*	0,07 *(0*,*20)*

One-way analysis of variance:

* p < 0.05,

** p < 0.01;

^1^ Welch test was applied due to nonhomogeneous variances across age groups.

For the young girls, NMT values for the hip abductors and adductors have not reached the peak strength values of young adult females. Starting with young adulthood, leg strength values decline with age. The elderly women (AG65) showed the smallest NMTs. These results correspond with previous studies analyzing lower extremity muscle strength differences between age groups. Starting at approximately 30 years of age and based on measured differences between young and older adults, interpolated average yearly strength declines of 1 to 1.5 percent across various body muscles are reported in reviews concerning an age-related strength loss [20,32,33].

The largest NMT values for the hip abductors were found in AG25 with Games-Howell post-hoc differences between the age groups all highly significant except for the difference between AG10 and AG50 (Table 3). The largest NMT values for the hip adductors were again found in AG25 with the Tukey post-hoc differences between the age groups all highly significant except for the difference between AG10 and AG50. The largest NMT values for the knee extensors were found in AG25 as well. Highly significant Tukey post-hoc differences were detected between AG10 and AG70, between AG25 and AG50 resp. AG70, and between AG50 and AG70. While declines in the average NMTs of the hip abductors and the hip adductors between AG50 and AG70 of 1.3 percent per year were detected, the average decline in the NMT values for the knee extensors between these age groups was only 1.0 percent.

**Table 3:** Significances of Tukey-HSD post-hoc tests for the group mean difference of the normalized maximal torques (lower left corner shaded in grey) in the knee extensors (KE), the hip abductors (AB), and the hip adductors (AD) as well as for the ratios (upper right corner) between hip abductors vs hip adductors vs knee extensors (AB/AD, AB/KE, AD/KE) in the four age groups (AG10 = 10- to 13-year old girls, AG25 = 19- to 29-year old females, AG50 = 41- to 55-year old females, and AG70 = 63- to 79-year old females).

Groups	AG10 (*n* = 31)	AG25 (*n* = 30)	AG50 (*n* = 27)	AG70 (*n* = 33)
AG10 (*n* = 31)		AB/KE**^1^, AD/KE**^1^	AB/AD*^1^	AB/AD*^1^
AG25 (*n* = 30)	AB**^1^, AD**		AB/KE*^1^, AD/KE**^1^	AB/KE**^1^, AD/KE**^1^
AG50 (*n* = 27)		AB**^1^, AD**, KE*		
AG70 (*n* = 33)	AB**^1^, AD**, KE**	AB**^1^, AD**, KE**	AB**^1^, AD**, KE**	

* p < 0.05,

** p < 0.01;

^1^ Games-Howell post-hoc test was applied due to nonhomogeneous variances across age groups.

**Fig 3:**
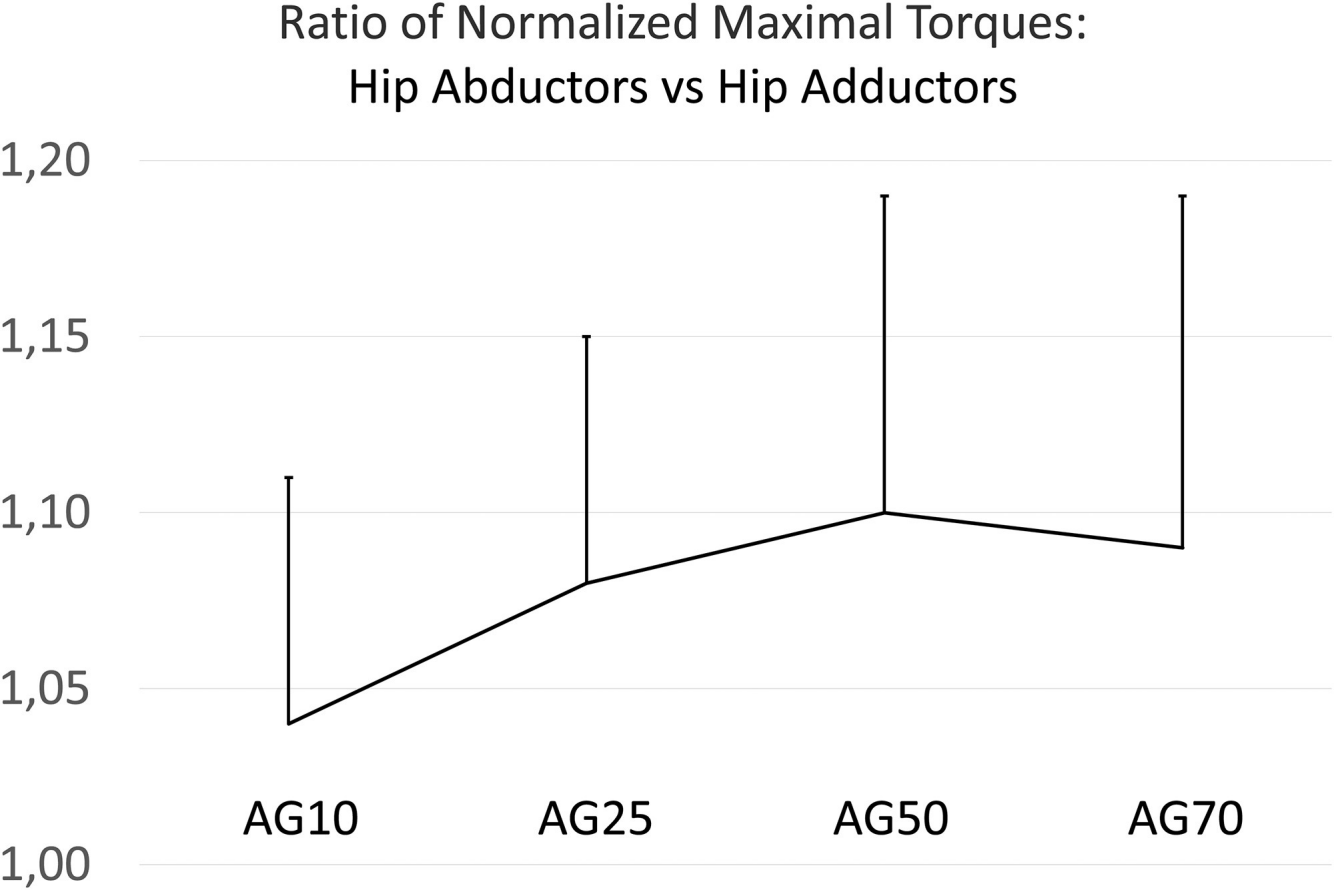
Ratios of normalized maximal torque ratios between the hip abductors and hip adductors in the four age groups (AG10 = 10- to 13-year old girls, AG25 = 19- to 29-year old females, AG50 = 41- to 55-year old females, and AG70 = 63- to 79-year old females).

To directly express the non-uniform strength declines in the stabilizer muscles versus the primary movers across age, the NMT ratios between hip abductor / hip adductors and the knee extensors were calculated for all groups. In Fig 2, a bell-shaped curvature for both ratios across age is visible. Since the homogeneity of variances in these ratios across age was not provided, the Welch test was applied. For this procedure, the differences in the NMT ratios between the hip abductors and the knee extensors across age groups were found to be highly significant (F (3,62.98) = 6.65 (8.24), p < 0.01; ω^2^ = 0.15). In addition, highly significant Games-Howell post-hoc tests were found between AG10 and AG25 and between AG25 and AG70. For the NMT ratio between the hip adductors and knee extensors, significant differences between the age groups were detected as well (F (3, 62.98) = 6.65 (8.01), p < 0.01; ω^2^ = 0.15). Pairwise, highly significant differences between the age groups were found in the Games-Howell post-hoc tests between AG10 and AG25, between AG25 and AG50, as well as between AG25 and AG70. For the analysis of the NMT hip abductor to the hip adductor ratio, the Levene test was found significant, indicating that homogeneity of variances was not provided as well. Nevertheless, in the oneway analysis of variance, a highly significant difference between the age groups was found for this ratio (F (3,64.13) = 4.36, p < 0.01; ω^2^ = 0.07). Games-Howell post-hoc differences were significant between AG10 and AG50 as well as between AG10 and AG70. Noteworthy, NMT ratios between hip abductors and hip adductors increase from childhood to the fifth life decade and appear to remain constant afterwards (Fig 3). Using the non-parametric Kruskal-Wallis test as an additional evaluation procedure, the statistical significances between the age groups reported above were confirmed.

## Discussion

In the following section, the study results will be discussed according to their compliance with related studies in the literature and their meaning for the elderly population in regard to their risk to fall. Next, consequences for strength training in the older adults will be outlined. Last but not least, a possible impact of the assessment and evaluation procedures will be reconsidered. The discussion will be ended by a critical analysis of the limitations of this study.

The goal of this investigation was to analyze the strength differences in leg stabilizer muscles versus primary movers in four female age groups. A hypothesis was tested stating that while older adults might use their leg stabilizers less frequently than their leg primary movers, different declines in the normalized maximal torques produced by these muscles should be observed across age groups. This hypothesis ties in with results by Trudelle-Jackson and coworkers [39] indicating non-uniform strength declines in lower extremity muscle groups across four female groups across 50 to 89 years of age. Their interpolated average yearly strength decrease in the hip abductors between 50 and 70 years of age was 1.2 percent as compared to 1.3 percent in our study. Interestingly, for the knee extensors, Trudelle-Jackson and colleagues found an average yearly strength decline of only 0.1 percent between these age groups as compared to 1.0 percent in our study. While average strength declines in the knee extensor muscle of approximately 1 percent were reported elsewhere in the literature [33, 59], a possible reason for this inconsistency may relate to the make-method in the hand-held measurements by Trudelle-Jackson and colleagues. To perform a make test with a hand-held device, examiners hold the dynamometer steady with one hand while manually stabilizing the client with the other hand. However, for knee angles of 90 degrees, measuring knee extensor strength with the make-method may be crucial with the examiner positioned in front of the subject and not above when using the body weight for a side-lying position to examine the hip abductors and adductors. In our study, this issue was addressed by conducting the knee extensor strength measurements with the examiner positioned with the back leaning against a wall. Prior to our study, we had analyzed the make-methode with a hand-held dynamometer with and without back support. Due to possibly large knee extensor strength, we decided to position the examiner with a back support while stabilizing the both elbows in front of the chest. In this position, one hand held the LMMTs towards the shank of the subjects with the other hand providing support.

Our knee extensor strength declines correspond with as well the values provided by Murray and coworkers [60]. They examined the isometric knee extensor strength at three knee angles (30, 45, and 60-degree knee flexion) comparing 20-year old to 86-year old women using a Cybex II dynamometer. Noteworthy, for the isometric 60-degree flexion measurements, their average yearly strength declines of 0.7 percent in the knee extensors when comparing young to old women, and 1.0 percent when comparing middle-aged to old women, were the same as in our study.

While our average strength declines in the hip abductors were consistent with the values of Trudelle-Jackson et al. [39] with both studies using with a hand-held dynamometer for a side-lying position, smaller declines for both the hip abductors and the hip adductors were previously reported in other studies [35,61] using isokinetic devices for isometric measurements in a standing upright position. The yearly decreases between early and late adulthood were in the range of 0.4 for the hip abductors and 0.7 percent for the hip adductors as compared to 1.0 percent for both muscle groups in our study. These conflicting findings show that care must be taken when using different measurement materials and subject positioning (standing upright vs. side-lying) to analyze strength decrease across age [62].

In the past, various reports have emphasized the particular importance of adductor and abductor strength in older adults for the maintainance of medio-lateral stability to prevent for falls [35,63,64,65]. Recently, Eckardt [66] showed that instability resistance training with free loads on unstable platforms, specifically activating stabilizer muscles [41], significantly improved medio-lateral gait stability on uneven surfaces while traditional resistance training of the leg extensors on stable platforms and isolated hip adductor and hip abductor training did not. Consistently, MacAulay et al. [67] found that spatial parameters in the gait pattern, rather than temporal parameters, separate fallers aged 60 and older from non-fallers in that same age category. Hence, more enhanced declines in the stabilizer muscles as compared to primary movers would indirectly imply an increased risk of falls in the older population. Assuming that elderly people are less exposed to daily activities stressing their leg stabilizers muscles than their leg primary movers, a more pronounced strength loss in the stabilizers would be expected. As a consequence, the risk of falls may increase. This argument is supported by results from Morcelli et al. [37]. These authors found significantly lower hip adduction, abduction, and extension strength in older fallers as compared to non-fallers. From their results, differences between both groups were more pronounced for the hip abductors and adductors with 12 and 13 percent than for the hip extensors with 9 percent. Similar results are provided in a recent study by Gafner and colleagues [68]. Our results support this view by showing less pronounced declines in the knee extensor strength values as compared to the stabilizer muscle groups (Fig 2). Other evidence in favor of our hypothesis comes from studies on the mechanisms of groin injuries in athletes. Strength deficits in stabilizing muscles of the hip and the pelvis, as compared to propulsive muscles, are considered to pose a risk factor for this injury type [69].

As smaller hip adductor / hip abductor to knee extensor strength values are found in older subjects, the question arises whether age and / or the lack of physical activity to be responble for this finding. An answer, in this regard, may be derived from our elderly subject group (AG65). Separating rather inactive from moderately active older subjects through a median split (at 60 min per week) on the self-reported physical activity times showed significantly smaller NMT values for the hip adductor, the hip abductor, and the knee extensor strength values in the rather inactive subjects. However, no significant differences were found between these subgroups for the hip adductor / hip abductor to knee extensor strength ratios while a slight tendency for smaller ratios in the rather inactive group existed. In contrast, no differences in the NMT values or their ratios were found between rather inactive and rather active subjects through a median split (at 120 min per week) on the self-reported physical activity times for the 41- to 55-year old females (AG50).

Given the particular importance of the hip stabilizer strength, resistance training for the adductor and abductor muscles has been recommended for the elderly population [38,70]. However, inconclusive results are found in the literature on which exercise provides best effects. For example, Delmore and co-workes [71] found larger peak and average emg activity of the adductor longus muscle in college students for the side-lying hip-adduction when compared to Swiss Ball squeezes, side lunges, standing adduction on a Swiss Ball, rotational squats, and sumo squats. In contrast, Serner and co-workers [72] detected smaller adductor longus muscle activation for the side-lying hip-adduction as compared to isometric adduction with a Swiss Ball between the knees, the Copenhagen adduction, hip adduction with an elastic band, sliding hip adduction exercise, and isometric adduction with a Swiss Ball between the ankles. Specific exercises for the gluteus maximus and medius have been examined by Reiman and co-workers [73]. As an alternative, instability resistance training may be applicable for elderly subjects as well [74]. Higher levels of leg and trunk stabilizer activation have been found in resistance exercises on unstable versus stable platforms [41]. Therefore, following the principle of resistance training specificity [75,76], resistance training on unstable platforms might be suitable to train the hip stabilizers as well. This training mode, when slowly introduced to the subjects, has been shown to be safe and effective for elderly subjects between 65 and 80 years of age [74]. In addition, it was recently shown that this leg strength exercise type significantly improved medio-lateral gait stability which is considered as an important risk of fall indicator. In contrast, traditional leg extensor strength training and isolated hip adductor and hip abductor strength training with machines on stable platforms did not show any substantial improvements [66].

The major limitations of this study concern the cross-sectional type findings and the validity of strength measurement method. In regard to the first issue, we cannot exclude that our cross-sectional type of findings may suffer from a random bias based on the subject selection. Although sample size estimation and a close-to-random recruitment of subjects were conducted, longitunal results are in need to further confirm our results. The second limitation concerns the lack of a gold standard to evaluate human muscle strength. Inconclusive findings based on measurements of young and old adults may evolve when comparing hand-held dynamometers and isokinetics devices. As pointed out above, different devices and measurement positions may influence the results though both ways to measure lower extremity muscle strength have repeatedly shown good reliability [51,68,77–80]. For example, Mentiplay and co-workers offers an extensive analysis incorporating inter-rater reliability, intra-rater reliability, and inter-device reliability for hand-held dynamometers. In contrast to reliability, validity appears to be a still open issue [77,81,82]. In particular, differences between studies in subject positioning, validation criteria, impact of experimenter strength, and measurement parameters may impede conclusive data interpretation. For example, hand-held measurements to analyze the reliability of knee extensor strength were conducted with subjects sitting up and with forces measured slightly above the talotibial joint line at a 90-degree knee angle [83] while other studies examined subjects in a prone-lying position at a knee angle of 35 degrees [81]. In both studies, validity was tested by comparing hand-held measurements to isokinetic dynamometry, leaving unanswered whether isokinetic measurements are a true gold standard or not. As an alternative, validity has been examined by the emg activation level in the hip abductor muscle when comparing a side-lying to a standing subject position [62]. For the analysis of hip muscle strength, subjects have been examined in supine positions [51,82], side-lying positions with different postural constraints in the opposite leg [68,70,84], or standing up [85]. In addition, different lever arms [84] and leg abduction angles during measurements [84,86] were examined in the course of strength assessments in the hip abductors and adductors. In particular, contradictory results evolve when analyzing the well-known hip abductor to hip adductor ratio in a side-lying versus a supine subject position [78,87] as an important indicator for possible groin injuries [69,88–94]. For example, Tyler et al. [88] conducted pre-season testing of hip muscles and knee extensors in Hockey Players with a hand-held device. Measurements for hip abduction and hip adduction strength were done with subjects in a side-lying position. Clearly larger hip abduction strength was found as compared to hip adduction strength. Pre-season hip adduction strength was 95 percent of abduction strength in the uninjured players but only 78 percent of abduction strength in the injured players. However, when comparing the side-lying to the supine subject positioning, Thorborg et al. [86] found larger hip abductor than hip adductor strength for supine position while larger hip adductor than abductor strength was found in the side-lying position.

There are two more methodological points that deserve particular notice. First of all, from the Thorborg and co-workers study, subject positioning must be considered when interpreting the hip abductor-to-adductor ratio to identify a risk of groin injury. Secondly, when analyzing hip adductor strength in a side-lying position, it is important to emphasize how the opposite leg is positioned. Thorborg et al. [79] as well as Gafner et al. [68] used a supporting bench for the opposite leg enabling subjects to exert larger hip adduction forces in the tested leg. As a result, in both studies larger hip adduction than hip abduction strength was detected. In turn, in our study using a side-lying subject position as well, no such support was provided. Therefore, smaller hip adduction than hip abduction forces were found. Noteworthy in our study is the evidence that adductor strength declines are increasingly more pronounced from childhood until approximately the sixth decade of life (Fig 3). In later adulthood, this trend levels off or even appears to be reversed. We assume that lower normalized strength values and NMT ratios found in the 10- to 13-year old girls, as compared to the adult subjects, could be related to physical development and maturation attributed to endocrine factors, general motor coordination, development of the nervous system [95], and muscle activation [96].

In addition to the above-mentioned issues of test validity, authors in the literature have indicated that when using the make-tests, the strength of the experimenter may influence the magnitude and the reliability of hand-held measurements [97,98]. As a consequence, for our knee extensor strength measurements with the LMMTS, the experimenter adopted a body positioning with back support close to a wall similar to Mentiplay et al. [51], providing sufficient resistance towards the subject’s force exertion. Previously, pilot measurements showed that higher reliability can be achieved using this experimenter positioning as compared to no back support. For the force measurements in the hip abductors and adductors, the experimenter bowed over the subjects with extended arms using all her body weight to support the isometric measurement. While keeping the arms extended, the experimenter was able to use her full body weight as resistive force during the strength measurements in the hip abductor and adductors. For a similar reason, a short lever arm was used although a longer lever arm has been reported to provide better reliability [84]. To avoid any influence of the experimenter strength, studies have assessed hip abductor and adductor strength with a dynamometer attached to a metal frame to provide isometric resistance [68].

Summarizing the above-mentioned measurement issues, care should be taken when interpreting absolute values of strength and intra-individual strength ratios. Consequently, various researchers have already emphasized that strength data with hand-held devices is strongly influenced by the measurement conditions [51,77,97–99]. While absolute values of leg strength and intra-individual strength ratios are hard to compare, our study aimed to show that leg strength measured in one and the same way differs across four different age groups. In contrast, many other studies (see reviews by Doherty [32] or Vandervoort [20]) identifying age-related strength declines referred to a comparison of only one young and one old subject group. In order to provide comparability by minimizing anthropometric influences related to the subjects, strength values were expressed relative to the body weight [35,38,54–56] and accounting for lever arm lengths [46,81,92,100] or body height [84]. Furthermore, subject instruction and verbal support were standardized across all measurements to restrict a possible bias provided by the experimenter [101,102]. Last but not least, we used the mean value of three measurements to increase reliability as compared to the best value [82].

In conclusion, our study revealed clear and highly significant differences in the NMTs between four age groups for the hip adductors, the hip abductors, and the knee extensors (Table 2), showing a close to bell-shape curvature in the NMT ratios across age (Fig 2). In particular, stabilizer muscle strength values showed a clearly more-pronounced decline when compared to primary mover strength. Hence, evidence was provided in this study implying a differential strength decline in the leg muscles in the course of the aging process. Moreover, analyzing four subject groups across a wide age range rather than comparing only young to old subjects may provide better insight of age-related decline in muscle strength [39,61]. As an important consequence, older adults should train their lower extremity muscle strength with a particular focus on stabilizer muscles. Instability strength training on unstable platform could be particularly beneficial for that purpose [41, 66, 74]. However, further studies are needed to confirm the age-related imbalance in the strength declines between leg muscles with different functional tasks. Possibly other subject positioning options and measurements with standardized isokinetic devices should be used to assess specific strength in primary movers and stabilizers across age groups not only in the lower extremities but in the upper extremities and the trunk as well. In order to use the ratio between stabilizer strength and primary mover strength as an indicator for the risk of falling in elderly individuals, specific measurement standards should be settled.
